# Static magnetic stimulation induces structural plasticity at the axon initial segment of inhibitory cortical neurons

**DOI:** 10.1038/s41598-024-51845-7

**Published:** 2024-01-17

**Authors:** J. L. Beros, E. S. King, D. Clarke, L. Jaeschke-Angi, J. Rodger, A. D. Tang

**Affiliations:** 1 School of Biological Sciences, The University of Western Australia, Crawley, 6009 Australia; 2 The Perron Institute for Neurological and Translational Science, Nedlands, 6009 Australia; 3 Département de Neurosciences, Université de Montréal, Quebec, H3C 3J7 Canada; 4 School of Biomedical Sciences, The University of Western Australia, Crawley, 6009 Australia

**Keywords:** Intrinsic excitability, Cellular neuroscience

## Abstract

Static magnetic stimulation (SMS) is a form of non-invasive brain stimulation that alters neural activity and induces neural plasticity that outlasts the period of stimulation. This can modify corticospinal excitability or motor behaviours, suggesting that SMS may alter the intrinsic excitability of neurons. In mammalian neurons, the axon initial segment (AIS) is the site of action potential initiation and undergoes structural plasticity (changes in length and position from the soma) as a homeostatic mechanism to counteract chronic changes in neuronal activity. We investigated whether the chronic application of SMS (6 and 48 h, 0.5 T) induces structural AIS plasticity in postnatally derived primary cortical neurons. Following 6 h of SMS, we observed a shortening in mean AIS length compared to control, that persisted 24 h post stimulation. In contrast, 48 h of SMS induced an immediate distal shift that persisted 24 h post-stimulation. Pharmacological blockade of voltage gated L/T-type calcium channels during stimulation did not prevent SMS-induced AIS structural plasticity. Our findings provide the foundation to expand the use of chronic SMS as a non-invasive method to promote AIS plasticity.

## Introduction

Non-invasive forms of brain stimulation that modulate neural activity or excitability have great potential as a tool to study neural plasticity mechanisms or as a treatment for neurological disorders. In particular, static magnetic stimulation (SMS) is a form of non-invasive brain stimulation where static magnetic fields are delivered to the brain by a rare-earth neodymium magnet placed at the surface of the skull^1^. In studies using human participants, SMS has been shown to modulate neural activity in the motor and sensory cortices^2–7^. In particular, delivering SMS for short periods (e.g., 10–30 min), most commonly leads to a reduction in cortical excitability and activity^3,4,6–9^, which may suggest that SMS leads to an acute increase in neural inhibition^5,8^. While many studies have focused on SMS and its immediate effects, more chronic applications of this intervention and its effect on neural plasticity and excitability are not known.

The axon initial segment (AIS) is the microdomain of the axon comprised of specialised anchoring and structural proteins and high densities of voltage-gated ion channels that facilitate AP initiation^10,11^. It is well established that the AIS of individual neurons undergoes structural remodelling (i.e. structural plasticity) during development^12–14^ and in response to significant periods of high and low neural activity^15,16^. Specifically, AIS structural plasticity includes changes in AIS length and starting position along the axon and this process is dependent upon the activity of voltage-gated calcium channels^10,17,18^. Such changes in AIS structure are believed to be a homeostatic response to perturbations in neural activity, as they have a direct functional consequence on neuronal excitability^10,13,17^. For example, in vitro and in vivo models have shown that significantly increasing neural activity via pharmacological or optogenetic interventions leads to a shortening of the AIS and/or a relocation to more distal start positions along the axon and vice versa for significant decreases in neural activity^13,15,16^.

Given that SMS is a simple method that can be used to alter neural activity, we sought to determine whether chronic SMS could be used to induce structural AIS plasticity of cortical neurons. We characterised the AIS structure of cortical neurons in vitro*,* following 6 or 48 h of SMS. Primary cortical neurons dissected from postnatal day 1 (P1) mice were cultured for 7 days in vitro (DIV7) and exposed to 0.5 Tesla (T) SMS. Cultures were processed for immunofluorescence to determine AIS length and starting position along the axon. We found that SMS induced an AIS shortening and/or distal relocation in inhibitory neurons after 6 and 48 h of stimulation, with some of these changes persisting to 24 h post-stimulation. Interestingly, the extent of these changes was similar to AIS structural plasticity induced with the chronic application of 15 mM potassium chloride (KCl), which is commonly used to increase activity and induce structural plasticity in vitro^15,18–20^. In an additional study, pharmacological blockade of L/T-type voltage-gated calcium channels did not prevent structural remodeling of the AIS after 6 h of SMS. Overall, our results demonstrate that chronic SMS induces AIS structural plasticity in inhibitory cortical neurons, suggesting that this may be a promising tool to induce AIS plasticity non-invasively.

## Results

**Figure 1:**
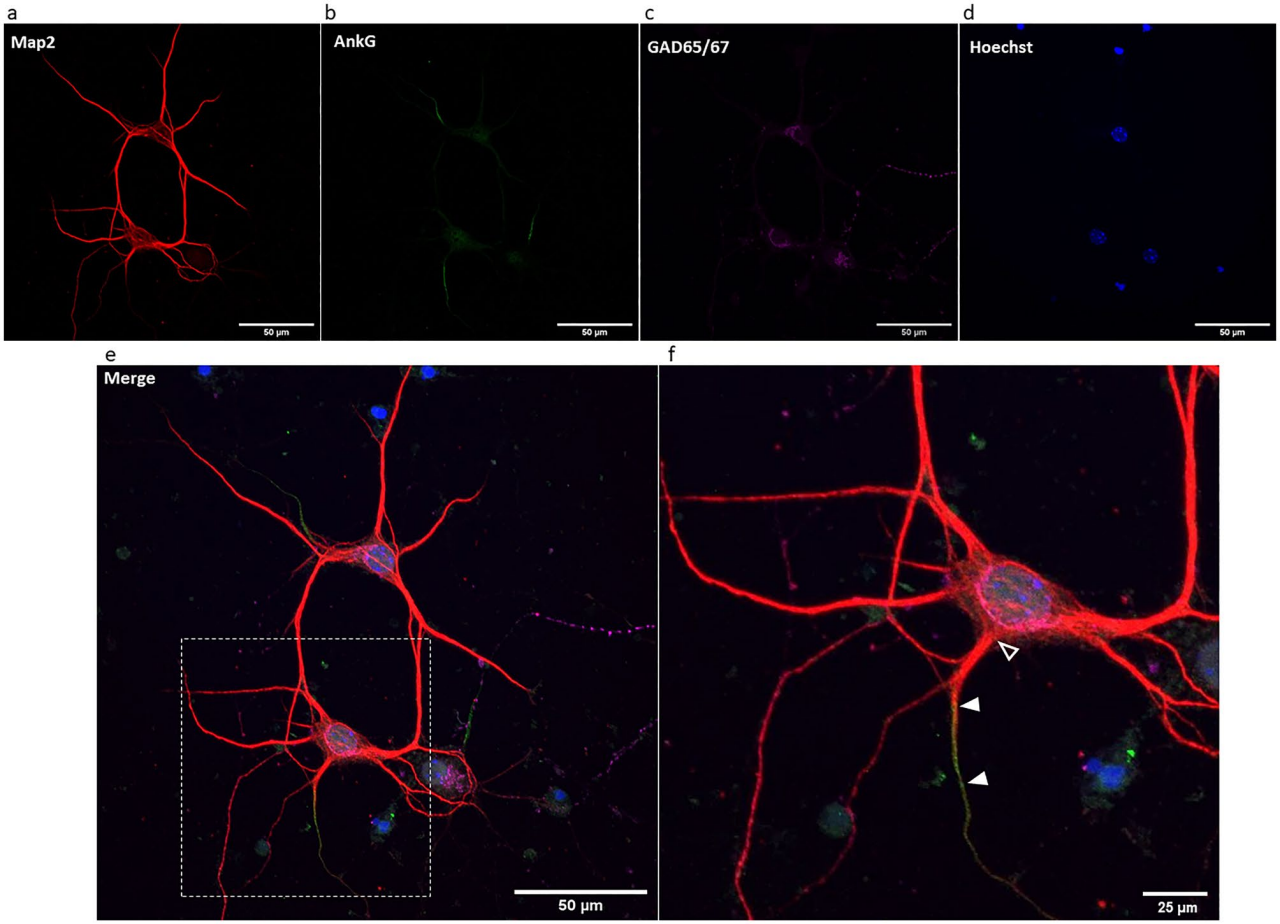
Maximum intensity z-projection of immunolabelled inhibitory cortical neurons in cell culture at DIV7. Neurons were immunolabeled for (**a**) the neuronal marker MAP2, (**b**) AIS marker AnkG, (**c**) inhibitory neuronal marker GAD65/67and (**d**) nuclear marker Hoechst. Images were (**e**) merged and AIS length and distance from soma were quantified. (**f**) A magnified image of the white box in (**e**). Filled triangles indicate the approximate start and end of the AIS and the unfilled triangle indicates the approximate edge of the soma used in AIS distance quantification. Images were captured on a C2 confocal microscope at 60 × magnification. Scale bars in (**a**–**e**) = 50 µm; (**f**) = 25 µm.

When testing for the identity of neurons in our cell cultures, the majority of MAP2 neurons present were immunopositive for the inhibitory marker GAD65/67 compared to the excitatory marker CaMKII (mean of ~ 83% GAD65/67 positive neurons, quantified from 5 control media coverslips from 5 culture experiments). As only a small population of neurons positive for microtubule-associated protein 2 (MAP2) did not express the inhibitory marker, we restricted our analysis to MAP2 + GAD65/67 immunopositive inhibitory neurons only. In these neurons, the AIS was identified by the immunofluorescence profile of AnkyrinG (AnkG; see Fig. 1), an anchoring protein highly expressed at the AIS and commonly utilised to identify the AIS and AIS structural plasticity^12,13,15,16^. We observed heterogeneity in AIS morphology in our cell cultures^19^, with the AIS originating both immediately proximal and more distal to the soma, indicating that our cultures contained a mixture of cells that had dendritic and somatic originating axons. All identified neurons were used in analyses regardless of AIS origin.

To determine cell viability in our coverslips, we added Image-IT™ viability stain to identify MAP2 positive neurons with compromised cell membranes indicative of cell death. When assessing neuron viability (Image-IT™-negative and MAP2-postive neurons) as a percentage of total MAP2-postive neurons in each coverslip, we found that the mean viability in our cultures ranged from ~ 77 to ~ 90%. We did not detect any differences between treatment groups or with increased DIV (Supplementary Fig. S1) which suggests that our treatments are not increasing cell death.

### KCl induced AIS structural plasticity in inhibitory neurons

**Figure 2:**
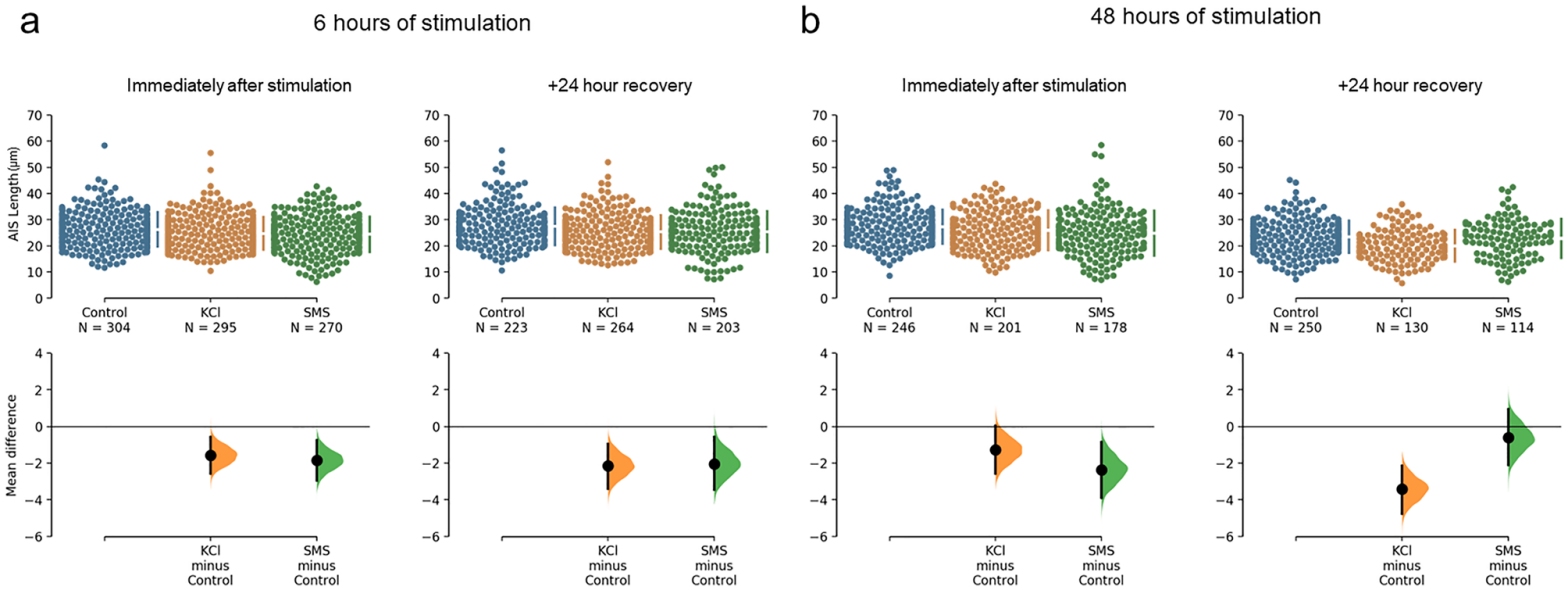
Cumming estimation plots of AIS lengths obtained from cultured cortical neurons treated with 15 mM KCl or 0.5 T SMS. (**a**) Raw data of AIS lengths obtained from individual neurons (top) and mean differences of treatment compared to the shared Control (bottom) plotted as bootstrap sampling distributions. Neurons were fixed immediately after (left panel) and 24 h after (right panel) 6 h of stimulation. (**b**) Raw data (top) of AIS lengths and mean differences (bottom) obtained immediately after (left panel) and 24 h after (right panel) 48 h of stimulation. The number of neurons analysed in each group are presented under the group names. Each mean difference in the bottom graph is depicted as a dot with 95% confidence intervals indicated by the vertical error bars. Data from each treatment group at each time point was collected from at least 5 coverslips over a minimum of 3 separate culture experiments.

**Figure 3:**
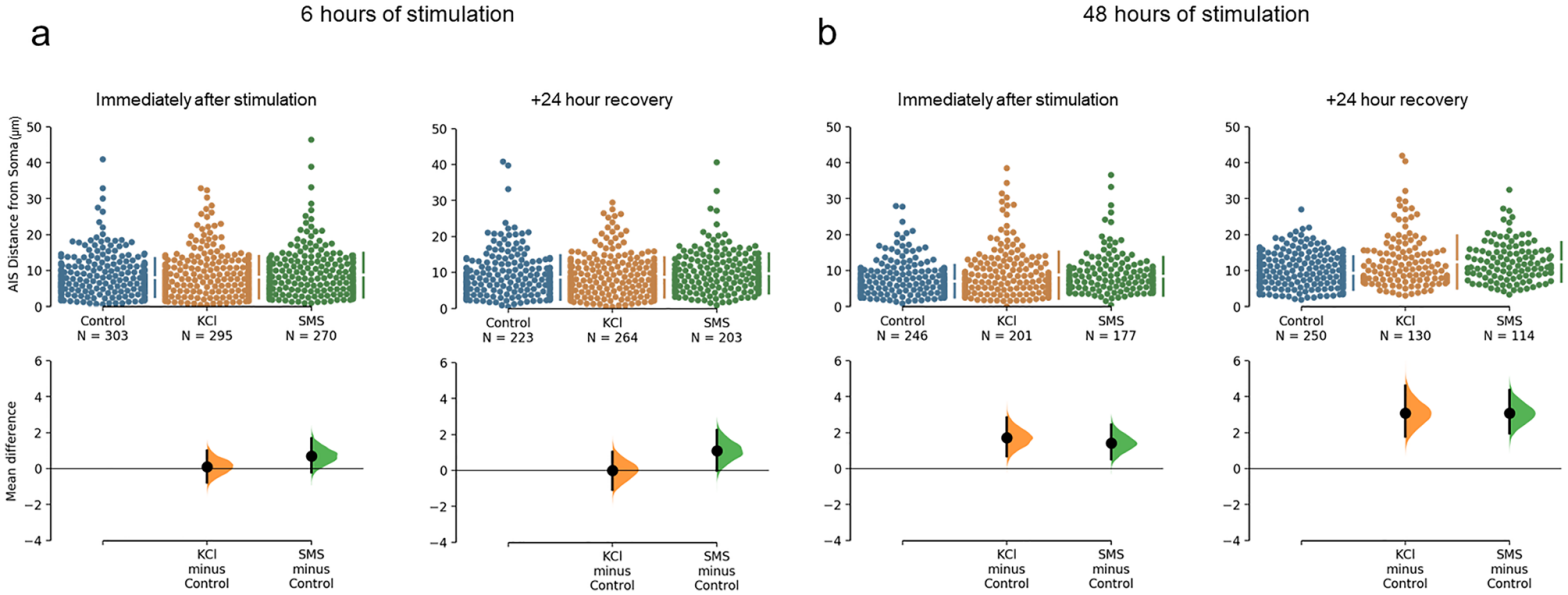
Cumming estimation plots of the proximal start of the AIS from the soma obtained from cultured cortical neurons treated with 15 mM KCl or 0.5 T SMS. (**a**) Raw data of AIS distance from the soma obtained from individual neurons (top) and the mean differences of treatment compared to the shared control (bottom) plotted as bootstrap sampling distributions. Neurons were fixed immediately after (left panel) and 24 h after (right panel) 6 h of stimulation. (**b**) Raw data (top) of AIS distance from the soma and mean differences (bottom) obtained immediately after (left panel) and 24 h after (right panel) 48 h of stimulation. The number of neurons analysed in each group are presented under the group names. Each mean difference in the bottom graph is depicted as a dot with 95% confidence intervals indicated by the vertical error bars. Data from each treatment group at each time point was collected from at least 5 coverslips over a minimum of 3 separate culture experiments.

We first confirmed that we could induce AIS structural plasticity in our postnatally cultured cortical neurons with 15 mM KCl applied to the cell culture medium. We found evidence that similar to embryonically derived primary neurons^15,21^, KCl induced structural plasticity of the AIS at most of our assessed time points (Figs. 2 and 3). After incubation for 6 h, the mean length of the AIS reduced from 26.28 ± 6.50 µm in control conditions to 24.71 ± 6.17 µm with KCl (Fig. 2a). The unpaired mean difference between these conditions was − 1.57 µm [− 2.57, − 0.58], representing a 6% reduction in AIS length. A Bayesian ANOVA indicated anecdotal evidence for a treatment effect (BF = 2.69), and post-hoc testing revealed posterior odds of 1.21 for a reduction in AIS length with KCl compared to control conditions. Interestingly, when cell cultures were returned to control media immediately after 6 h of KCl stimulation and fixed 24 h later, the mean AIS length remained shortened when compared to neurons in the control condition (27.44 ± 7.12 µm in control and 25.28 ± 6.30 µm in KCl; Fig. 2a). This represented an unpaired mean difference of -2.15 µm [− 3.39, − 0.95] and ~ 8% reduction in AIS length. Results from the Bayesian ANOVA for AIS length indicated moderate evidence for a treatment effect (BF = 6.41) with posterior odds of 24.37 between control and KCl conditions, suggesting strong evidence for a continued reduction in AIS length 24 h after 6 h of stimulation.

When stimulating neurons for 48 h, the mean AIS length of KCl treated neurons was 25.96 ± 7.61 µm compared to 27.24 ± 6.41 µm in control conditions (Fig. 2b). The mean difference between these conditions was − 1.28 µm [− 2.57, 0.05] and we found no evidence for a treatment effect on AIS length (BF = 0.18) at this time point. However, we did see a delayed reduction in AIS length when cell cultures were returned to control medium 24 h after this 48 h stimulation period. The mean AIS length of neurons in control conditions at this time point was 23.42 ± 6.15 µm compared to 19.99 ± 6.05 µm with KCl. This resulted in a mean difference of − 3.43 µm [− 4.77, − 2.14], ~ 15% reduction in AIS length (Fig. 2b). These results were supported with our Bayesian analysis revealing extreme evidence for a treatment effect (BF = 736.75), and post-hoc testing revealing posterior odds of 19,846.32 which suggests extreme evidence for a delayed reduction in AIS length with KCl stimulation.

When assessing for changes in the proximal start of the AIS from the soma, after 6 h of stimulation the mean distance in neurons treated with KCl was 8.16 ± 5.88 µm which was similar to control neurons with a mean of 8.05 ± 5.36 µm (Fig. 3a). This represented a mean difference of 0.11 µm [− 0.78, 0.98] with no evidence of a main effect on AIS distance (BF = 0.02). We also found no evidence for any delayed changes in AIS distance when cell cultures were returned to control medium for 24 h after 6 h of stimulation (BF = 0.05). The mean AIS distance of KCl stimulated neurons (8.61 ± 5.55 µm) was similar to neurons that remained in the control conditions (8.60 ± 6.16 µm; mean difference < 0.01 µm).

However, in cells that received 48 h of KCl stimulation, the mean proximal start position of the AIS was located more distal (8.72 ± 6.53 µm) when compared to neurons in control medium (7.00 ± 4.50 µm). This was a distal relocation with a mean difference of 1.72 µm [0.70, 2.83], or ~ 25% more distal compared to neurons in control conditions (Fig. 3b). This was supported with a Bayesian ANOVA demonstrating extreme evidence of a treatment effect on AIS distance at DIV10 (BF = 910.96) with anecdotal evidence for a distal shift with KCl compared to control medium (posterior odds of 2.89). This mean distal shift in the AIS with 48 h of KCl stimulation persisted for 24 h after returning neurons to control medium (9.30 ± 4.62 µm in control and 12.39 ± 7.30 µm with KCl). This was a mean difference of 3.09 µm [1.79, 4.59], or ~ 23% distal relocation (Fig. 3b). Results from the Bayesian ANOVA indicated extreme evidence for a treatment effect at DIV10 (BF = 106.56) with posterior odds of 1624.87 indicating extreme evidence for this prolonged distal relocation of KCl treated neurons compared to control.

### SMS induced AIS structural plasticity in inhibitory neurons

We found that 0.5 T SMS applied to neurons for 6 h resulted in a reduction in mean AIS length to 24.44 ± 6.68 µm, a mean difference of − 1.84 µm [− 2.95, − 0.76] and 7% reduction compared to neurons in the control condition (Fig. 2a). Bayesian ANOVA revealed strong evidence for this difference with posterior odds of 52.34. This reduction in AIS length persisted 24 h after stimulation ceased (mean AIS length of 25.40 ± 7.79 µm) with a mean difference of -2.04 µm [− 3.44, − 0.58] or ~ 7.5% reduction compared to control neurons. There was anecdotal evidence for this difference with posterior odds of 2.90. Immediately after 48 h of SMS we witnessed a reduction in the mean AIS length of − 2.36 µm [− 3.88, − 0.86] when compared to control neurons but we found no evidence of a treatment effect (BF = 0.18). There were also no delayed changes in AIS length when neurons were returned to control medium for 24 h following 48 h of stimulation; the mean AIS length of neurons in the SMS condition was 22.82 ± 7.37 µm, a mean difference of − 0.60 µm [− 2.10, 0.93] compared to control neurons.

In addition to these changes in the length of the AIS after SMS, we also found evidence for a distal relocation in the mean start position of the AIS relative to the soma with longer stimulation times (Fig. 3). After 48 h of SMS, the mean proximal start of the AIS increased from 7.00 ± 4.50 µm from the soma in control medium to 8.43 ± 5.33 µm with SMS. This was a mean difference of 1.43 µm [0.51, 2.44; Fig. 3b] or ~ 20% distal relocation with strong evidence for this difference (posterior odds of 27.52). This persisted when SMS ceased and neurons were returned to control medium for 24 h (9.31 ± 4.62 µm in control and 12.40 ± 5.49 µm with SMS). The resulting mean difference was 3.09 µm [1.96, 4.36] or ~ 33% distal relocation, with extreme evidence for this effect (posterior odds of 128,025.71). Our results suggest that similar to KCl stimulation, chronic SMS induced a structural change in the AIS of cortical neurons and that this effect outlasted the period of stimulation.

### Blocking L/T type voltage gated calcium channels does not prevent SMS induced AIS structural plasticity

**Figure 4:**
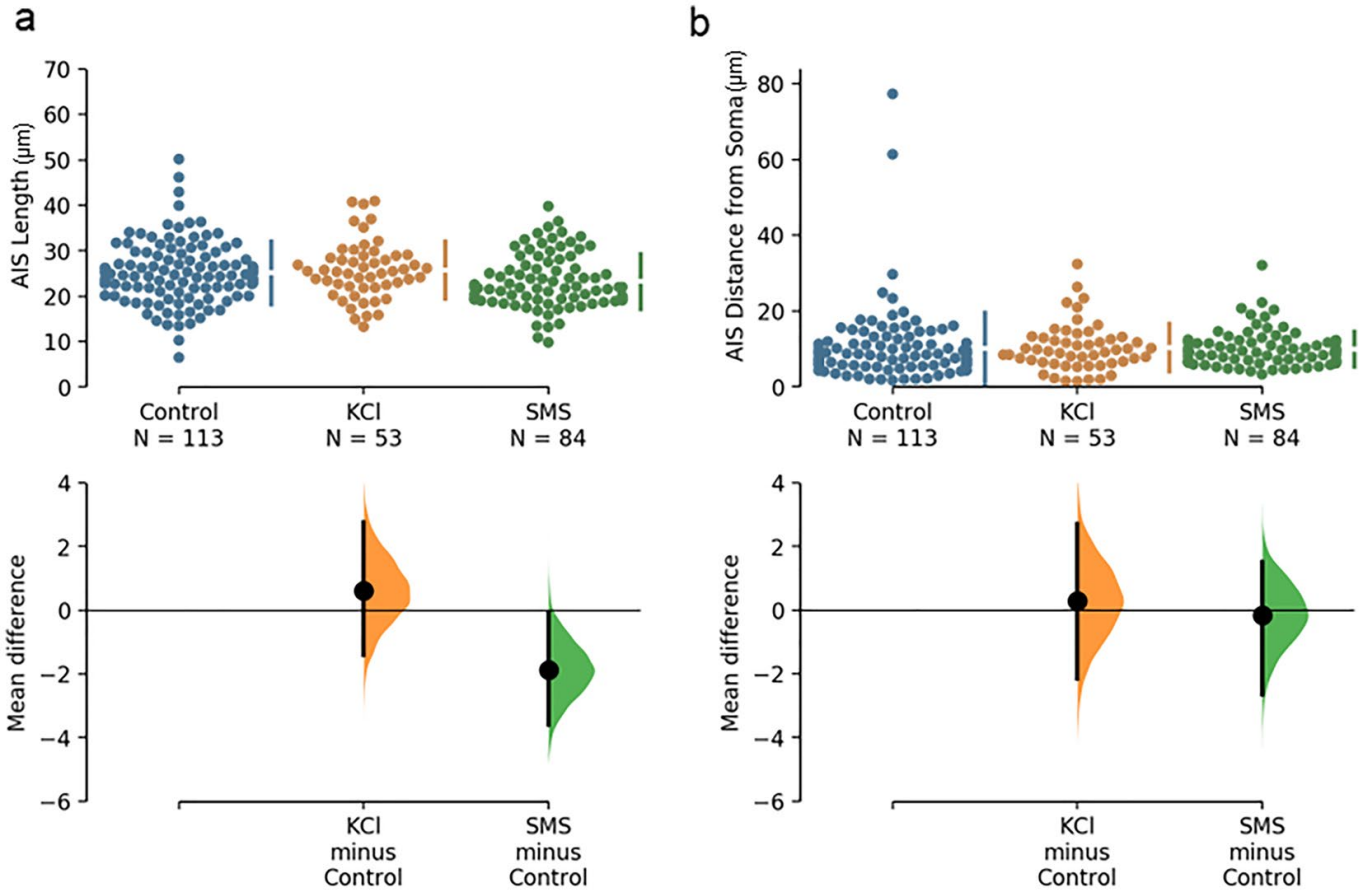
Quantification of AIS length and distance from soma immediately after 6 h of stimulation with 3 µM of mibefradil dissolved in the culture medium of all treatment groups. (**a**) Cumming plots of AIS length and (**b**) AIS distance from soma. Raw data obtained from individual neurons (top) and the mean differences of treatment compared to shared control (bottom) plotted as bootstrap sampling distributions. Number of neurons analysed in each group presented in brackets under the group names. Data from each treatment group was collected from at least 3 coverslips from 3 separate culture experiments.

To assess if SMS-induced AIS plasticity occurs through similar mechanisms as KCl-induced AIS plasticity, we added 3 µM of the L/T type voltage-gated calcium channel blocker mibefradil during the 6 h stimulation period. Comparing mean AIS lengths in the presence of mibefradil for both KCl (25.73 ± 6.32 µm) and SMS (23.25 ± 6.01 µm) stimulated neurons to the control treatment (25.11 ± 6.92 µm; Fig. 4a) revealed no evidence of a main effect of mibefradil (BF = 0.23) or treatment*mibefradil interaction (BF = 0.09). The length of the AIS of neurons in the SMS condition remained shortened when compared to neurons in the control condition with an unpaired mean difference of − 1.87 µm [− 3.59, − 0.07], indicating that mibefradil did not prevent SMS induced changes in AIS length.

When assessing AIS distance from the soma, we found anecdotal evidence (BF = 1.37) that the addition of mibefradil was associated with a distal relocation that was observed across all treatment groups when compared with treatment groups that were not incubated with mibefradil. We did not find any evidence of an interaction effect of mibefradil with our treatment groups (BF < 0.01) and the distances of the AIS from the soma were similar in KCl (10.39 ± 9.45 µm) and SMS (9.94 ± 4.67 µm) conditions compared to control neurons (10.09 ± 9.45 µm; Fig. 4b).

## Discussion

Here, we demonstrate the novel finding that chronic application of 0.5 T SMS induces AIS structural plasticity in inhibitory cortical neurons in vitro. We found that in postnatally derived primary cortical cultures, KCl induces reductions in AIS length and AIS distal relocations that was prevented with the application of 3 µM of mibefradil. Similar to the changes induced by 15 mM KCl, we found strong evidence that 0.5 T SMS delivered for 6 h resulted in a reduction in AIS length and this persisted for 24 h. We found strong evidence that stimulating neurons with SMS for 48 h induced a relocation in the proximal start of the AIS distal to the soma that persisted 24 h after stimulation ceased.

Our results show that similar to studies using embryonically derived neurons in cell culture, sustained depolarisation of postnatally derived inhibitory cortical neurons in cell culture with 15 mM KCl induces a shortening and/or distal relocation of the AIS^15,18,21^. These changes were on a similar scale to the changes seen with KCl stimulation of cultured olfactory bulb GABAergic dopaminergic interneurons^20^. Interestingly, we found that the AIS of inhibitory cortical neurons did not return to control lengths and locations after returning the neurons to control medium for 24 h after 6 and 48 h of stimulation. It was observed in the previously mentioned study using olfactory bulb GABAergic dopaminergic neurons that AIS lengths did not return to control values until 5 days post-stimulation^20^. While our neurons were obtained at a later age compared to those used in that study, our experiments were conducted earlier following seeding and may exhibit a slightly different developmental response in AIS structural plasticity^12–14^. Nevertheless, these results provide further evidence that structural AIS plasticity in inhibitory neurons is relatively slow to return to baseline.

As seen in the KCl treatment group, 0.5 T SMS induced AIS structural plasticity in inhibitory neurons when delivered for 6 and 48 h (i.e. chronic stimulation). It is thought that acute static magnetic fields interact with the diamagnetic anisotropic properties of membrane phospholipids and reorient membrane ion channels^22^, and can impede sodium and calcium channel kinetics^23,24^**.** Given that previous studies have shown that AIS shortening and distal relocation occurs following “excitatory” forms of stimulation^15,20^, this suggests that chronic SMS may be a form of excitatory stimulation. These findings contrast with the acute application of SMS (2–30 min) which generally depresses cortical excitability and neural activity^2–7,9^. The effect of chronic periods of SMS are not well described in the literature and the effect of SMS on neural activity (e.g. inhibition or excitation) may be dependent on stimulation time and intensity. While the reduction in AIS length and distal relocation following SMS would suggest a corresponding decrease in neuronal excitability, our quantification was purely structural and electrophysiological studies are needed to confirm functional AIS plasticity, although recent modelling experiments suggest that small AIS length changes of approximately 2 µm affect action potential properties that may decrease neuron excitability^25^. It is also important to note that neurons in our cell culture preparations were mostly immunohistochemically positive for GAD65/57 rather than CaMKII. It has been demonstrated that cultured excitatory hippocampal neurons in certain circumstances can be immunopositive for the GAD protein, suggesting that GAD may be post-transcriptionally regulated and excitatory neurons may exhibit phenotypic plasticity^26^. Therefore, while our work provides a foundation of SMS-induced AIS plasticity, future work is needed to characterise cell type specific SMS-induced AIS plasticity, particularly in an in vivo setting where the balance of excitation and inhibition is critical for neural circuit function.

One of the key findings from our experiments was that SMS induced both immediate and delayed effects on AIS structural plasticity which varied between 6 and 48 h of SMS. We found strong evidence that the 6 h SMS protocol caused changes in AIS length while 48 h of stimulation induced a distal relocation. These results suggest that in inhibitory neurons, the duration of SMS can be used to regulate the extent of AIS plasticity, with shorter time periods (i.e. 6 h) more appropriate for reducing AIS lengths and longer applications (i.e. 48 h) driving distal relocations. This adds to the body of work showing that the method of AIS structural remodeling (i.e. length and distance changes) can be induced independently of each other while resulting in the same functional outcome of changing excitability. The benefit of inducing changes to either AIS length or location is unclear as the plasticity mechanisms that separate remodeling of AIS length from location are not yet known. Therefore, the choice of SMS protocol as a tool to induce AIS may be better based on the magnitude of the changes induced, which would make 48 h of SMS more suitable as it induced larger AIS remodeling compared to 6 h of stimulation. This identifies SMS as a potential useful tool to study these mechanisms, as by altering stimulation duration, it may be possible to preferentially induce a change in either AIS length or distance.

In embryonically derived hippocampal excitatory neurons, KCl or optogenetic stimulation in the presence of mibefradil prevents AIS structural plasticity and any associated changes in neuron excitability^15^, suggesting that elevations in neuronal activity induce AIS plasticity through the activation of L/T-type voltage gated calcium channels. Surprisingly, application of mibefradil during stimulation did not prevent SMS-induced changes to AIS length immediately after 6 h of stimulation, suggesting that SMS is recruiting AIS plasticity independently of L/T-type voltage gated calcium channels. It has recently been shown in organotypic slice cultures of the avian nucleus cellularis^27^ that multiple voltage gated calcium channels induce AIS plasticity, all of which converge on the activation of extracellular-regulated kinase (ERK). Therefore, we speculate that SMS may be recruiting AIS plasticity through the activation of other voltage gated calcium channels (e.g.,P/Q type), as 1 h of 0.75 T SMS has previously been suggested to increase intracellular calcium and ERK through the activation of voltage gated ion channels in rat cortical neurons^28^. Future work would benefit from evaluating the role of other calcium channels (e.g., P/Q type), as it is known that multiple calcium voltage gated ion channels are involved in AIS plasticity^27^.

To determine whether SMS-induced AIS plasticity was due to increased cell death or injury, a known driver of AIS plasticity^29,30^, we quantified neuron viability in our cell cultures at the end of our stimulation periods. We found that the viability of neurons in our cell cultures did not seem to vary with any of our treatments (Supplementary Fig. S1), indicating that SMS or KCl for periods of 6 or 48 h in culture does not induce neuronal death greater than control conditions. Another important aspect of our study was the use of Bayesian statistics (Bayesian mixed factor ANOVA) to analyse AIS plasticity, which we believe allows for more meaningful interpretations of AIS structural plasticity. Specifically, as the changes in AIS structure are often in the order of a few microns, quantifying the amount of evidence for the observed effect makes the interpretation of seemingly small changes more intuitive than a p-value^31^.

In conclusion, we found that 0.5 T SMS can induce structural plasticity of the AIS in postnatal derived cortical neurons. Shorter stimulation periods (6 h) induced reductions in AIS length while longer applications (48 h) induced a distal relocation away from the soma. Our results demonstrate that as an experimental tool in vitro, chronic SMS may provide a cheaper and simpler alternative to other methods that are used to drive AIS plasticity (e.g. optogenetics) and may be a useful approach to further probe the mechanisms of AIS plasticity.

## Methods

### Preparation and maintenance of cortical cell cultures

P1 wildtype C57Bl/6 J mice were delivered from the Animal Resource Centre (Murdoch, Western Australia) to the University of Western Australia. Animals were euthanised with an overdose of sodium pentobarbitone (Lethabarb, Virbac) administered by an intraperitoneal injection. Animal procedures were approved by the UWA Animal Ethics Committee prior to commencement (RA/3/100/1677) and all experiments were performed in accordance with the Australian code for the care and use of animals for scientific purposes. After confirmation of death, the cortices of each mouse were dissected into serum free medium consisting of 2% B27 plus supplement (Gibco; Catalog number A3582801), 0.25% GlutaMAX supplement (Gibco; Catalog number 35050061) in Hibernate A medium (Gibco; Catalog number A1247501). The meninges were carefully removed from the dissected cortices, which were then cut into smaller pieces (approx. 0.5 mm^2^) using a scalpel blade. Cortices were equilibrated at 30 °C for 10 min prior to transferring them into pre-warmed dissociation medium consisting of 2 mg/ml papain (Sigma-Aldrich) and 0.25% Glutamax (Gibco) in Hibernate A medium without calcium and magnesium (BrainBits) that was pre-filtered through a 0.2 µm filter. This mixture was spun at 350 rpm at 30 °C for 30 min, after which, the cortices were transferred into 2 ml of pre-warmed dissection medium. Cortices were triturated using a sterile glass Pasteur pipette (Corning) and the supernatant was transferred into a 15 mL centrifuge tube. After repeating the trituration process and supernatant removal for an additional two times, the collected supernatant was centrifuged at 12,000 rpm for 5 min. The supernatant was aspirated and the pellet was resuspended in culture medium consisting of 2% B27 plus supplement (Gibco), 0.25% Glutamax (Gibco) and 0.1% penicillin/streptomycin (Gibco; Catalog number 15140163) in Neurobasal Plus medium (Gibco; Catalog number A3582901). Cell counts were estimated using a haemocytometer and seeded at a density of 150,000 cells/coverslip onto 13 mm diameter coverslips in 24 well plates (Trajan) pre-coated with 50 µg/ml poly-d-lysine (Gibco; Catalog number A3890401) and 40 µg/ml mouse type 1 laminin (Gibco; Catalog number 23017015). Cell culture plates were incubated at 37 °C with 5% CO_2_ for a period of 7 days before stimulation, with 50% of the media replaced at DIV4.

### Stimulation of cell cultures

On the morning of DIV7, 50% of the cell culture medium was replaced from each well prior to treatment with pharmacological (positive control), SMS or control. Pharmacological stimulation was achieved by dissolving 15 mM of potassium chloride (KCl; Sigma Aldrich) into the cell culture medium, previously shown to increase neuronal activity in culture and induce morphological changes in the AIS within 6 h of stimulation^15^. SMS was delivered by mounting the culture plate and coverslip on a north-facing 13 mm diameter rare earth neodymium magnet (0.5 T intensity at surface of the magnet) within the incubator. Control cultures did not receive any stimulation protocol and were maintained in normal culture medium in the incubator for the stimulation times. To assess the immediate effects of our stimulation protocols, the stimulation interventions were applied for 6 or 48 h and fixed immediately post-stimulation. These stimulation times have been previously observed to induce structural AIS plasticity and functional changes in excitability in cell culture and slice preparations^15,18,21^. To assess any delayed effects following stimulation, after the completion of the 6 and 48 h stimulation periods in a separate cohort of cultures, 90% of the cell culture medium was replaced in each well and cells were fixed 24 h later. In an additional study to investigate the molecular mechanisms underlying SMS induced AIS plasticity in our neuron population, we pharmacologically blocked L/T-type voltage-gated calcium channels with mibefradil (Abcam; product number 120343). 3 µM of mibefradil was dissolved into the culture medium 1 h prior to stimulation and maintained during the 6 h stimulation period for all treatment groups. This concentration has previously been shown to prevent AIS plasticity and any resulting changes in neuron excitability in cell culture^15,18,21^. Prior to cell culture fixation, culture medium was removed from all wells, washed twice with sterile phosphate buffered saline (PBS; Gibco; Catalog number 20012027) and fixed with 4% paraformaldehyde in 0.1 M PBS for 8 min. After the fixation period, wells were washed twice with 0.1 M PBS and stored in 0.1 M PBS with 0.1% sodium azide. To quantify cell viability in a separate cohort of cell cultures, 30 min prior to the end of the 6 h stimulation period, we added 1 µl of Image-IT™ DEAD Green™ Viability Stain (Invitrogen; Catalog number 10291), an impermanent dye used to identify dying cells. After the 30 min incubation and completion of the stimulation period, cell cultures were washed twice with PBS, fixed and stored as described above.

### Immunohistochemistry

Cell culture wells were washed twice with 0.1 M PBS followed by permeabilisation with 0.2% Triton-X in 0.1 M PBS for 10 min. Following this, cultures underwent a blocking step with 10% normal goat serum (ThermoFisher) in 0.2% Triton-X in PBS for 1 h. To identify neurons, cells were immunolabelled for microtubule-associated protein 2 (MAP2), expressed in the soma and dendrites of neurons, in addition to glutamic acid decarboxylase 65/67 (GAD65/67), a marker of inhibitory neurons expressed in the soma and processes. For AIS analysis, we paired the neuronal markers with antibodies against ankyrin G (AnkG), an anchoring protein highly expressed at the AIS and commonly utilised to identify the AIS and AIS structural plasticity^12,13,15,16^. Cultures were incubated in primary antibodies for mouse anti-AnkG (1:500, Thermofisher; catalogue number 33-8800; RRID: AB_2533145), rabbit anti-GAD 65/67 (1:1000, Thermofisher; catalogue number PA5-36080; RRID: AB_2553361) and chicken anti-MAP2 (1:5000, Abcam; catalogue number ab5392; RRID: AB_2138153) in a solution of 2% normal goat serum in 0.2% Triton-X in 0.1 M PBS at 4 °C for 24 h. After primary antibody incubation, wells were washed three times with 0.1 M PBS and then incubated with secondary antibodies goat anti-mouse Alexa Fluor 488 (1:600, Invitrogen), goat anti-rabbit Alexa Fluor 594 (1:600, Invitrogen) and goat anti-chicken Alexa Fluor 647 (1:600. Invitrogen) in the same primary blocking solution for 2 h at room temperature. The nuclear label Hoechst (1:1000, Invitrogen) was included in the secondary antibody incubation as a non-specific cell marker. Following this, wells were washed three times with 0.1 M PBS and coverslips were mounted on glass slides (Hurst Scientific) with Prolong™ diamond antifade mountant (Life Technologies).

### Imaging and cell quantification

After a minimum of 48 h, coverslips were imaged on a C2 confocal microscope (Nikon) at 60 × magnification (oil immersion, NA = 1.40, Nikon Plan Apo). Images were acquired in NIS Elements (Nikon) at a resolution of 512 × 512 pixels. The total area of the coverslip was divided into thirds and 15 fields of view were acquired in a systematic random manner (5 fields of view from each third). This represented a total sample area of approximately 0.5% of each coverslip. Multiple images were captured in the z-plane of each field of view, at a z-step depth of 0.125 μm, capturing the appearance and disappearance of all AIS fluorescence in the field of view. Images were processed in FIJI (Image J) to generate a single RGB TIFF of the maximum z projection. Prior to analysis, investigators were blinded to the stimulation group that the images belonged to.

AIS length was quantified using a custom Matlab code developed by Grubb and colleagues^15^. Briefly, the axon is manually traced using the AnkG fluorescence intensity, with the start and end position of the AIS defined as the first and last point along the axon where the AnkG fluorescence profile diminished to 0.33 of the maximum fluorescence value^18^. Cells that showed branching of the AIS/AnkG were excluded from the analysis. The start position of the AIS relative to the soma was estimated using Fiji image processing software by measuring the distance between the end of the MAP2 fluorescence signal and the start of AnkG fluorescence. Cells were classified as inhibitory neurons if they showed positive co-labelling for MAP2, GAD65/67 and DAPI. Each neuron was considered a separate data point and the sample size was collected over multiple coverslips over several culture runs (see figure legends). A power analysis was not conducted prior to project commencement. For cell viability quantification, in each coverslip 9 fields of view were uniform random sampled at 20 × magnification. Investigators were blinded to the identity of the images and counts were made of Hoechst-positive cells (total cells), MAP2 positive cells (neurons) and MAP2 positive cells with Image-IT™ in the nuclear region as per manufacturer instructions. One coverslip from each treatment group was analysed in 3 separate cell culture runs and coverslips that contained less than 50 Hoechst positive cells over all summed fields of view were not included in the final analysis.

### Statistical analysis

AIS data collected from each neuron was considered an independent sample, however we also report the number of coverslips and cultures that the data was obtained from. We conducted shared-control estimation statistics to determine differences in KCl and SMS treatment groups from the control condition. Cumming plots are presented detailing raw values from each treatment group in addition to the mean differences from the control condition. Mean data for treatment groups are presented as mean ± 1 standard deviation, while mean differences are presented as mean difference [95% confidence interval]. Estimation statistical analysis and generation of figures was obtained using resources described by Ho et al.^32^.

To assess if there was evidence for AIS plasticity, we conducted a Bayesian ANOVA. For the 6 h time point, we conducted a two-way Bayesian mixed factor ANOVA with treatment group (control, KCl and SMS) and drug (with and without mibefradil) as fixed factors and the culture run they were obtained from as a random factor. For the other assessed time points, a one-way Bayesian mixed factor ANOVA was conducted with treatment group (control, KCl and SMS) as a fixed factor and culture run as a random factor. In this way our statistical model computes the effect of our fixed factors on each neuron, while also considering random variance that may be the result of differences between culture runs. Bayesian analysis provides several advantages over frequentist analysis (i.e. generation of p values), including the generation of Bayes Factors that report how much evidence there is for the null or alternative hypothesis. Using this analysis, the amount of evidence for, or lack of an effect can be interpreted more intuitively (for a greater description on the use of Bayesian statistics in neuroscience, see Keysers et al.^33^). A custom prior value of 0.5 was used in our analysis and evaluation of a range of prior values (0.1–0.9) showed that our Bayes Factors (BF) were not sensitive to extreme values. BFs are reported according to cut-offs that suggest evidence in favour of the alternative hypothesis: BF ≤ 1 = no evidence, 1 < BF ≤ 3 = anecdotal, 3 < BF ≤ 10 = moderate, 10 < BF ≤ 100 = strong, BF < 100 = extreme. Where there was evidence for a treatment effect, Bayesian post-hoc testing based on pairwise comparisons using Bayesian t-tests was conducted with prior odds set at 0.5. All posterior odds were corrected for multiple testing by multiplying the uncorrected BF by the prior odds. Descriptive statistics of the mean and 95% credible intervals are presented in Supplementary Fig. S2 to supplement the analysis. Bayesian analysis was conducted using JASP software (Version 0.17.3) and data of AIS distance from all timepoints was log transformed prior to analysis to conform with statistical assumptions of normality (visual inspection of Q-Q plots).

### Supplementary Information

Supplementary Figures.

## Data Availability

Datasets will be provided upon request to the corresponding authors.

## References

[1] Nojima I, Oliviero A, Mima T (2020). Transcranial static magnetic stimulation: From bench to bedside and beyond. Neurosci. Res..

[2] Oliviero A (2011). Transcranial static magnetic field stimulation of the human motor cortex. J. Physiol..

[3] Kirimoto H (2014). Effect of transcranial static magnetic field stimulation over the sensorimotor cortex on somatosensory evoked potentials in humans. Brain Stimul..

[4] Dileone M, Mordillo-Mateos L, Oliviero A, Foffani G (2018). Long-lasting effects of transcranial static magnetic field stimulation on motor cortex excitability. Brain Stimul..

[5] Silbert BI, Pevcic DD, Patterson HI, Windnagel KA, Thickbroom GW (2013). Inverse correlation between resting motor threshold and corticomotor excitability after static magnetic stimulation of human motor cortex. Brain Stimul..

[6] Shibata S (2021). Effects of transcranial static magnetic stimulation over the primary motor cortex on local and network spontaneous electroencephalogram oscillations. Sci. Rep..

[7] Aguila J, Cudeiro J, Rivadulla C (2014). Effects of static magnetic fields on the visual cortex: Reversible visual deficits and reduction of neuronal activity. Cerebral Cortex.

[8] Davila-Pérez P, Pascual-Leone A, Cudeiro J (2019). Effects of transcranial static magnetic stimulation on motor cortex evaluated by different TMS waveforms and current directions. Neuroscience.

[9] Lozano-Soto E (2017). Transcranial static magnetic field stimulation (tSMS) of the visual cortex decreases experimental photophobia. Cephalalgia.

[10] Grubb MS (2011). Short- and long-term plasticity at the axon initial segment. J. Neurosci..

[11] Kole, M. H. P. & Stuart, G. J. Signal processing in the axon initial segment. *Neuron* **73**, 235–247. 10.1016/j.neuron.2012.01.007 (2012). 10.1016/j.neuron.2012.01.007 22284179

[12] Gutzmann, A. *et al.* A period of structural plasticity at the axon initial segment in developing visual cortex. *Front. Neuroanat.* **8**. 10.3389/fnana.2014.00011 (2014). 10.3389/fnana.2014.00011 PMC3949221 24653680

[13] Jamann N (2021). Sensory input drives rapid homeostatic scaling of the axon initial segment in mouse barrel cortex. Nat. Commun..

[14] Akter N, Fukaya R, Adachi R, Kawabe H, Kuba H (2020). Structural and functional refinement of the axon Initial segment in avian cochlear nucleus during development. J. Neurosci..

[15] Grubb MS, Burrone J (2010). Activity-dependent relocation of the axon initial segment fine-tunes neuronal excitability. Nature.

[16] Kuba H, Oichi Y, Ohmori H (2010). Presynaptic activity regulates Na+ channel distribution at the axon initial segment. Nature.

[17] Yamada, R. & Kuba, H. Structural and functional plasticity at the axon initial segment. *Front. Cell. Neurosci.* **10**. 10.3389/fncel.2016.00250 (2016). 10.3389/fncel.2016.00250 PMC5078684 27826229

[18] Evans MD (2013). Calcineurin signaling mediates activity-dependent relocation of the axon initial segment. J. Neurosci..

[19] Höfflin, F. *et al.* Heterogeneity of the axon initial segment in interneurons and pyramidal cells of rodent visual cortex. *Front. Cell. Neurosci.* **11**. 10.3389/fncel.2017.00332 (2017). 10.3389/fncel.2017.00332 PMC5684645 29170630

[20] Chand AN, Galliano E, Chesters RA, Grubb MS (2015). A distinct subtype of dopaminergic interneuron displays inverted structural plasticity at the axon initial segment. J. Neurosci..

[21] Evans MD, Dumitrescu AS, Kruijssen DLH, Taylor SE, Grubb MS (2015). Rapid modulation of axon initial segment length influences repetitive spike firing. Cell Rep..

[22] Rosen AD (2003). Mechanism of action of moderate-intensity static magnetic fields on biological systems. Cell Biochem. Biophys..

[23] Rosen AD (2003). Effect of a 125 mT static magnetic field on the kinetics of voltage activated Na+ channels in GH3 cells. Bioelectromagnetics.

[24] Rosen, A. D. Inhibition of calcium channel activation in GH3 cells by static magnetic fields. *Biochim. Biophys. Acta (BBA) Biomembranes* **1282**, 149–155. 10.1016/0005-2736(96)00053-3 (1996). 10.1016/0005-2736(96)00053-3 8679652

[25] Jungenitz, T. *et al.* Structural plasticity of the axon initial segment in rat hippocampal granule cells following high frequency stimulation and LTP induction. *Front. Neuroanat.* **17** (2023). 10.3389/fnana.2023.1125623 PMC10113456 37090138

[26] Cao Y (1996). Presence of mRNA for glutamic acid decarboxylase in both excitatory and inhibitory neurons. Proc. Natl. Acad. Sci. U S A.

[27] Jahan, I., Adachi, R., Egawa, R., Nomura, H. & Kuba, H. CDK5/p35-dependent microtubule reorganization contributes to homeostatic shortening of the axon initial segment. *J. Neurosci.* JN-RM-0917-0922. 10.1523/JNEUROSCI.0917-22.2022 (2022). 10.1523/JNEUROSCI.0917-22.2022 PMC9864565 36639893

[28] Prina-Mello A, Farrell E, Prendergast PJ, Campbell V, Coey JMD (2006). Influence of strong static magnetic fields on primary cortical neurons. Bioelectromagnetics.

[29] Vascak M, Sun J, Baer M, Jacobs KM, Povlishock JT (2017). Mild traumatic brain injury evokes pyramidal neuron axon initial segment plasticity and diffuse presynaptic inhibitory terminal loss. Front. Cell. Neurosci..

[30] Buffington SA, Rasband MN (2011). The axon initial segment in nervous system disease and injury. Eur. J. Neurosci..

[31] van Doorn J (2021). The JASP guidelines for conducting and reporting a Bayesian analysis. Psychon. Bull. Rev..

[32] Ho J, Tumkaya T, Aryal S, Choi H, Claridge-Chang A (2019). Moving beyond P values: Data analysis with estimation graphics. Nat. Methods.

[33] Keysers C, Gazzola V, Wagenmakers E-J (2020). Using Bayes factor hypothesis testing in neuroscience to establish evidence of absence. Nat. Neurosci..

[34] Percie du Sert N (2020). The ARRIVE guidelines 20: Updated guidelines for reporting animal research. PLOS Biol..

