# Supplementary material for: Static magnetic stimulation induces structural plasticity at the axon initial segment of inhibitory cortical neurons
Source: Sci Rep. 2024 Jan 17;14:1479. doi: 10.1038/s41598-024-51845-7 (PMC10794225; doi:10.1038/s41598-024-51845-7)
Supplement: Supplementary file 1 — Supplementary Figures. [file 41598_2024_51845_MOESM1_ESM.pdf]

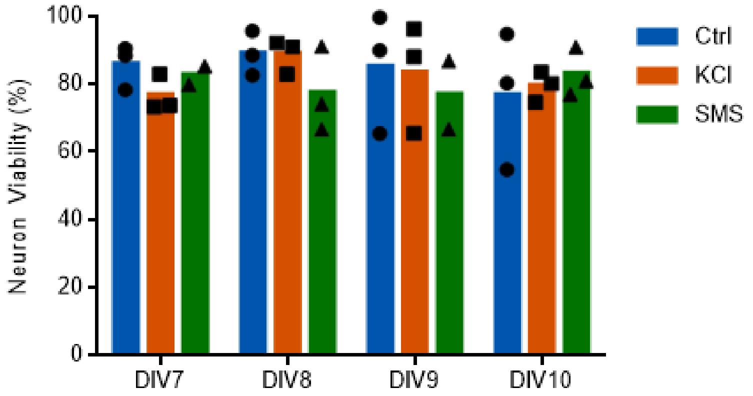

Supplementary Figure 1. Neuron viability in cell cultures at different time points using Image-IT™ DEAD Green™ Viability Stain. (b) Data is presented as MAP2-postive neurons with no nuclear Image-IT™ as a percentage of total MAP2-postive neurons. Mean values from individual coverslips collected over 3 separate culture runs are plotted as black dots (9 fields of views collected from each coverslip). Coverslips with less than 50 total cells over all obtained images were not included in the analysis.

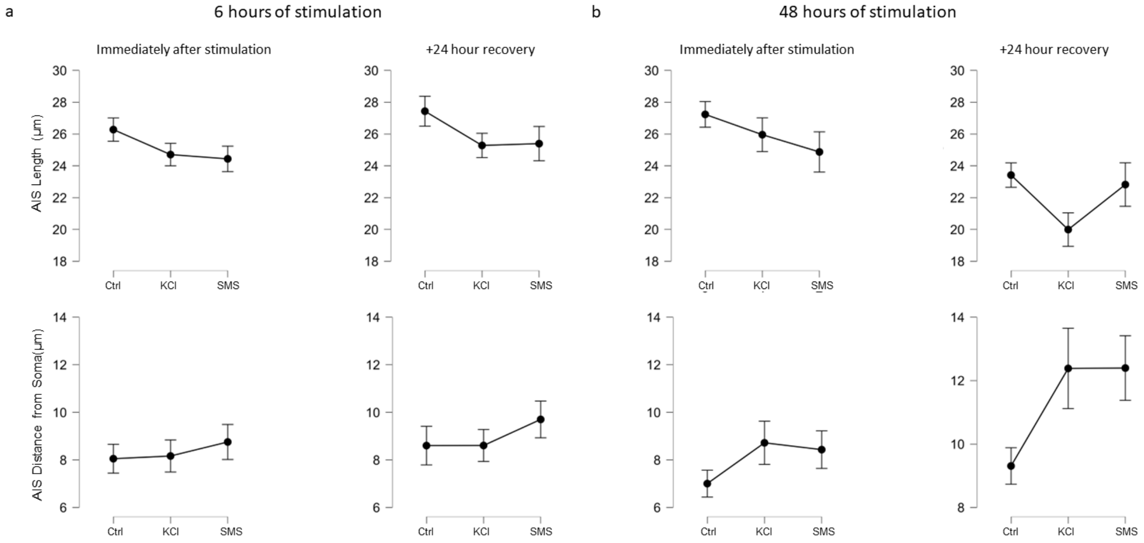

Supplementary Figure 2. Mean values of AIS measurements for each treatment group at all time points. Mean AIS length (top panel) and proximal start of the AIS from soma (bottom panel) for (a) 6 hours of stimulation and (b) 48 hours of stimulation. Black bars represent the 95% credible interval.
